# Detection of adeno-associated virus type 2 genome in cervical carcinoma

**DOI:** 10.1038/sj.bjc.6603179

**Published:** 2006-05-30

**Authors:** B Y Zheng, X D Li, F Wiklund, S Chowdhry, T Ångstrom, G Hallmans, J Dillner, K L Wallin

**Affiliations:** 1 Department of Molecular Medicine and Surgery, Karolinska Institute, Center for Molecular Medicine, Karolinska University Hospital Solna, Stockholm 171 76, Sweden; 2 Oncology Center, Umeå University Hospital, Umeå, Sweden; 3 Cytology Laboratory, Umeå University Hospital, Umeå, Sweden; 4 Department of Public Health and Clinical Medicine, Nutritional Research, Umeå University Hospital, Umeå, Sweden; 5 Department of Medical Microbiology, Lund University, Malmö University Hospital, Malmö, Sweden

**Keywords:** adeno-associated virus, AAV, cervical cancer, human papillomavirus, HPV

## Abstract

Adeno-associated virus (AAV) can impair the replication of other viruses. Adeno-associated virus seroprevalences have been reported to be lower among women with cervical cancer. *In-vitro*, AAV can interfere with the production of human papillomavirus virions. Adeno-associated virus-2 DNA has also been detected in cervical cancer tissue, although not consistently. To evaluate the role of AAV infection in relation to invasive cervical cancer, we performed a nested case–control study within a retrospectively followed population-based cohort. A total of 104 women who developed invasive cervical cancer on average 5.6 years of follow-up (range: 0.5 months–26.2 years) and 104 matched control-women who did not develop cervical cancer during the same follow-up time were tested for AAV and human papillomavirus by polymerase chain reaction. At baseline, two (2%) case-women and three (3%) control-women were positive for AAV-2 DNA. At the time of cancer diagnosis, 12 (12%) case-women and 3 (3%) matched control-women were positive for AAV-2 DNA. Persisting AAV infection was not evident. In conclusion, AAV-2 DNA was present in a low proportion of cervical cancers and we found no evidence that the presence of AAV in cervical smears of healthy women would be associated with reduced risk of cervical cancer.

Cervical cancer ranks as the second most common cancer among women worldwide. Human papillomavirus (HPV) infection has been established as the main causal factor for cervical cancer (Munoz *et al*, 2003; Schiffman and Castle, 2003). During carcinogenesis, integration of high-risk HPV causes imbalance in the expression of the viral oncoproteins E6 and E7 which degrades tumour suppressor genes p53 and retinoblastoma protein (Rb), respectively (Alazawi *et al*, 2002). Proposed cofactors to HPV in cervical carcinogenesis include other sexually transmitted infections such as *Chlamydia trachomatis* infection (Koskela *et al*, 2000), smoking and genetic risk factors (Ghaderi *et al*, 2002), but these have not fully explained why some HPV-exposed women develop cancer and others do not.

Adeno-associated virus is a helper-dependent parvovirus that needs co-infection with other viruses, such as adenovirus (Atchison *et al*, 1965; Henry, 1973), HPV (Hermonat, 1994; Su and Wu, 1996; Hermonat *et al*, 1997), vaccinia virus (Schlehofer *et al*, 1986) or herpes simplex virus (HSV) (Salo and Mayor, 1979; Buller *et al*, 1981) to help its replication. Among all AAV types, AAV-2 is the common cause of infection in humans (Friedman-Einat *et al*, 1997). Adeno-associated virus-2 Rep78 gene inhibits HPV-induced cell transformation *in vitro* (Hermonat, 1994; Hermonat *et al*, 1997; Zhan *et al*, 1999), thus suggesting the inhibition of HPV gene expression by AAV-2. However, Ahn *et al* (2006) could not show any significant viral inhibition in all cervical cancer cell lines by the AAV-2 Rep 78 gene.

An inverse association between seropositivity for AAV-2 and cervical cancer has been reported (Mayor *et al*, 1976; Georg-Fries *et al*, 1984). However, studies on detection of AAV DNA in cervical smears or biopsies do not fully support the serological findings. Several studies detected AAV DNA in cervical samples (Han *et al*, 1996; Venturoli *et al*, 2001; Ahn *et al*, 2003), while others failed to detect AAV DNA in cervical samples (Strickler *et al*, 1999; Odunsi *et al*, 2000). Coker *et al* (2001) found that AAV is associated with reduced risk in high-grade squamous intra epithelial lesion (HSIL), but not low-grade squamous intraepithelial lesion (LSIL). However, Ahn *et al* (2003) found that AAV is not associated with all stages of cervical precancer and invasive cancer lesions by *in situ* hybridisation and immunohistochemistry.

Whether presence of AAV DNA is associated with cervical cancer risk has not been assessed in an epidemiological controlled format. The aim of this study was to investigate if AAV-2 infection was associated with decreased or increased risk for future development of cervical cancer, using a retrospective, population-based study design.

## MATERIALS AND METHODS

### Study base

The study base consisted of the women residing in the Västerbotten county in northern Sweden who attended the population-based four-yearly interval invitational cervical screening programme. The population in the county was 260 472 inhabitants of whom 130 651 were women in 1995. The programme invites women in the age group of 25–59 years to participate and it has an attendance rate of >80% since it was introduced in 1969. All cervical cancer patients are treated at the Umeå University Hospital and all clinical data are stored in the same hospital. Similarly, all cytological diagnoses are made and all specimens stored at the Cytology Laboratory, Umeå University Hospital (Wallin *et al*, 1999).

### Study design

The study was based on an earlier case–control retrospective study as described in Wallin *et al* (1999). Eligible case-women had been diagnosed with invasive cancer between the years 1969–1995 after having had at least one normal smear taken, had at least one additional smear (irregardless of its cytological diagnosis) taken and had not had any operative treatment of the cervix. A total of 133 eligible case-women were identified by linking the cytological registry with the Swedish National Cancer Registry. Further linkage to the pathological registry was made for the retrieval of histopathological samples for re-review and laboratory analysis. Four women were excluded due to incorrect entries into the cancer registry, 12 due to missing histopathological specimens and 11 due to non-invasive cervical neoplasia, leaving 106 cases of invasive cervical cancer. Of these two biopsies were inadequate for polymerase chain reacion (PCR) analysis, leaving 104 cases (85 squamous cell carcinoma and 19 adenocarcinoma), with serial samples available for the study. Control subjects were women from the same study base who did not develop cervical cancer during the same period before the diagnosis of cancer in their corresponding cases. Each control was matched one to one to its case according to age and time of sampling of the baseline smears and existence of normal follow-up smears matched in time of sampling to the cervical cancer biopsies. Thus, each case-woman contributed a normal smear taken before invasive cancer diagnosis and a histological sample with a confirmed diagnosis of cervical carcinoma, and each control woman contributed two normal smears matched to the normal smear and biopsy of its corresponding case. The mean time between the sampling of the baseline smear and cancer diagnosis was 5.6 years (range: 0.5 months–26.6 years). The average age at which the baseline smear was obtained was 44 years (range: 19–74) among the women with cancer and 44 years (range: 20–74) among the control-women. The dates of the smears of the women with cancer and those of the control-women differed by 1 month, on average. The subsequent smears of the control-women were typically obtained after the diagnosis of cancer in the corresponding women with cancer, as absence of disease in the controls for at least the same duration of follow-up was an essential component of the study design. If a control-woman had several normal smears after the date of the diagnosis of cancer in the corresponding woman with cancer, the smear obtained on the date closest to the date of the diagnosis of cancer was chosen. The time of biopsy in the woman with cancer and the time the smear was obtained in the corresponding control-woman differed by up to 10 months. The average age of the women with cancer at the time of diagnosis was 50 years (range: 24–79), and the average age of the control-women at the time of the second normal smear was 50 years (range: 24–79) (Wallin *et al*, 1999).

All cytological smears and histological slides were re-evaluated before laboratory analysis. Ethical approval was obtained from the Institutional Review Board of Umeå University. As decided by the Institutional Review (IRB): Umeå University (2003-02-11, 98/03, Dnr 98-12), information about the study was made known to the public that donated the samples by press conferences.

### DNA extraction from paraffin sections and archival Pap smears

DNA was extracted from archival smears and biopsies as described earlier (Chua *et al*, 1996). In brief, coverslips of Pap smears were removed with xylene and the cytoplasmic stain was removed in 95% ethanol. A mild lysis buffer containing 25 *μ*l of proteinase K (20 mg ml^−1^) (Sigma, Sigma-Aldrich, Hamburg, Germany) per 1.0 ml of buffer was used to dislodge the cells off the glass slide, and the cells were pipetted into a sterile Eppendorf tube. An additional 10 *μ*l of proteinase K was added and digestion was carried out at 55–60°C for a minimum of 2 h. Protein was precipitated by the addition of 100 *μ*l saturated ammonium acetate, and centrifuged at 14 000 rpm at room temperature for 5 min. DNA was precipitated using cold ethanol and dissolved in low EDTA, tris-EDTA (TE) buffer.

Four 5 *μ*m-thick sections were taken from each paraffin block. Knives were changed and an empty paraffin block sectioned in between each biopsy to prevent cross-contamination. The fifth section was stained with haematoxylin and eosin for review to ensure that the proceeding sections contained tumour tissue for PCR analysis. Deparaffinization was carried out in xylene and the tissue rehydrated through graded alcohols, then air-dried and digested with buffer containing 10 *μ*l proteinase K (20 mg ml^−1^). Digestion was carried out at 60°C for a minimum of 2 h until a clear lysate was obtained. Heating the samples at 98°C for 10 min inactivated proteinase K.

All samples were tested for DNA integrity by PCR using human ribosomal gene S14 primers, which gave 150 bp amplimers. The blank sections were tested by S14 PCR to check for contamination during sectioning.

### Adeno-associated virus polymerase chain reaction

As positive control, DNA extracted from HA-16 cells (kindly donated by Professor JR Schlehofer) was used. It is a cervical cancer cell line HeLa (containing HPV 18) transfected with AAV-2 (Walz and Schlehofer, 1992). These cells were grown in DMEM medium (PAA laboratories GmbH, Austria) supplemented with 10% fetal calf serum (FCS), streptomycin (50 mg ml^−1^) and penicillin (500 U ml^−1^) and DNA was extracted for PCR. DNA was extracted according to the standard protocols and measurements were made by UV-1601spectrophotometer (Shimadzu, Japan).

Three primer pair sets were chosen for AAV detection. (1) The pan 1/pan 3 primer pairs are general primers for AAV-2, -3 and -5 which have a size of 338 bp (Tobiasch *et al*, 1998). A sensitive panel was set up for pan AAV PCR, in which total HA-16 DNA concentration varied at 50, 100, 250 and 500 pg. Negative controls for AAV consisted of DNA extraction from HeLa cell line, and blanks containing no DNA were also included. The detection limit for the pan-AAV primers PCR system was 100 pg total HA-16 DNA. (2) Non-nested AAV-2 primers (Han *et al*, 1996) and (3) AAV-nested PCR (Mehrle *et al*, 2004), both AAV-2 PCR systems, target the AAV-2 *rep* gene. They were tested against a sensitive panel total HA-16 DNA with DNA concentration ranging from 230 ng to 0, 23 pg. The detection limit for non-nested AAV-2 PCR system was 23 pg while that of nested AAV-2 was 2, 3 pg. Oligonucleotide primers used in this study are detailed in Table 1.

Optimisation of the PCR reactions was tested at different annealing temperatures, primer concentrations and MgCl_2_ concentrations. DNA amplification was performed in a 50 *μ*l reaction mix containing 1.0–3.0 *μ*l of template DNA, and for second round nested PCR, 5 *μ*l of the first round of PCR product was used as template DNA, 1 × PCR buffer, 1.5–2.0 mM MgCl_2_, 0.2 mM of each of dATP, dCTP, dTTP, dGTP (Roche, Germany), 10 pmol of each of pan-AAV or AAV-2 primers, respectively, 5 *μ*l of 2% BSA and 1.0–1.5 U Taq DNA polymerase (Promega, Madison WI, USA). Polymerase chain reactions were performed using Peltier Thermol Cyclerb PTC-100 (MJ Research, Inc. Waltham, MA, USA). The PCR reaction consisted of 40 cycles with the denaturation step at 94°C for 30 s, followed by the annealing step at 62°C for pan-AAV or 64°C for non-nested AAV-2 or 62°C for nested AAV-2 PCR, respectively, for 30 s and extension at 72°C for 45 s. Each PCR was initiated by one denaturation step at 95°C for 1 min before the amplification cycles and completed by one extension step at 72°C for 5 min after 40 amplification cycles. A volume of 10 *μ*l PCR products were loaded into 2% agarose gel and visualised with ethidium bromide. Positive PCR products were sequenced according to the protocol for ABI Prism™ BigDye™ Terminator Cycle Sequencing Ready Reaction Big Dye Terminators Kit kit (Perkim Elmer Applied Biosystems, Foster City, CA, USA). Blanks containing no DNA were included as contamination controls. Varying amounts of template DNA was used on repeated PCR runs to overcome the effect of inhibitors present in the DNA extraction.

To test the specificity of the AAV PCR systems, different PCR primer sets directed towards pan-AAV, AAV-2 (nested or non-nested) and HPV amplification were tested on DNA from HA-16 cells (contain HPV 18 and AAV-2), SiHa cells (contain HPV 16) and HeLa cells (contain HPV18).

### Human papillomavirus polymerase chain reaction

The HPV consensus primers MY09/MY11 and GP5+/6+ were used in single-tube nested PCR, where the GP5+/6+ products were amplified in abundance for gel electrophoresis and HPV typing by DNA sequencing. Details of the procedure had been published (Wallin *et al*, 1999). The PCR analyses were performed blind to cases or controls and were analysed at the same time, to avoid the risk of assay performance variation. The sensitivity of the PCR system was based on dilutions of HPV-16 L1 plasmids (1 copy to 500 copies) mixed with 2 *μ*l of DNA extracts from 10 HPV PCR-negative cervical smears as a check for PCR inhibition. Dilutions of (1 fg–10 pg) DNA extracts from HPV-16-positive SiHa cervical cancer cell-line were also included into each PCR run as a sensitivity panel. DNA from HPV-16-positive CaSki and HPV-negative C33A cervical cancer cell-lines were also included as controls (Wallin *et al*, 1999).

### HPV DNA typing

Human papillomavirus typing was performed first with type-specific primers for HPV-16 (E1: 1768–1960) and HPV-18 (E7: 591–900) as described (Evander *et al*, 1991). Samples that were HPV-positive by general primers but negative by type-specific primers were subjected to DNA sequencing of the GP5+/6+ amplimers according to the ABI Prism™ BigDye™ Terminator Cycle Sequencing Ready Reaction Big Dye Terminators Kit (PE Applied Biosystems, Perkim-Elmer Corp., Foster City, CA, USA) and sequenced using the ABI 310 sequencer (PE Applied Biosystems).

### Statistical analysis

Relative risk with 95% confidence interval (CI) was estimated as odds ratio (OR) by logistic regression using epiinfo software (Epi Info 2000, Centers for Disease Control and Prevention, Atlanta, Georgia, USA).

## RESULTS

The PCR primer specificity test showed a positive band using the pan-AAV and the two AAV-2 PCR systems with HA-16 DNA but not when SiHa or HeLa cell DNA was used as template. HPV general primers gave a positive band when all three cervical cancer cell-lines were tested, which is expected as they all contain HPV DNA.

The level of sensitivity for the three PCR systems used in this study was 2.3 pg HA-16 DNA for nested AAV-2, 23 pg for non-nested AAV-2 and 100 pg for pan-AAV. The detection limits were repeatable and consistent in each PCR run for each system. The S14 PCR, which was incorporated to test the amplifiability of DNA, was positive in all samples tested in this material. To overcome possible intrinsic inhibition of PCR amplifications, three different rounds of PCR of the entire study material were repeated using 1–3 *μ*l of template DNA.

For pan-AAV PCR, AAV DNA was not detectable in any of the baseline smears and their corresponding matched controls, nor in the cervical cancer biopsies. The non-nested AAV-2 PCR system detected AAV DNA in one cervical cancer biopsy and one control smear matched to a cancer biopsy. With the nested AAV-2 PCR, 2/104 (2%) of the prediagnostic smears of cancer cases and 3/104 (3%) of the prediagnostic smears of matched control-women were positive for AAV DNA. At cancer diagnosis, 12/104 (11.7%) of biopsies with cancer were positive and 3/104 (3%) of matched control smears to the biopsies were positive for AAV DNA (OR for cancer in case of AAV DNA positivity: 4.39 (95% CI: 1.11–20.29). Persisting AAV infection in the same woman was not found in any one of the women in our study (Table 2). Results of the HPV DNA testing have been reported previously (Wallin *et al*, 1999). In brief, HPV DNA status at baseline among cytologically normal smears for cases was 29.8% (31/104) as compared to 2.8% (3/104) among controls. Seventy-seven percent (80/104) had HPV DNA in the cervical cancer biopsies. Of these case-women, 27 had persistent HPV DNA at both the baseline smear and the cancer biopsy, while only one control-woman was HPV DNA-positive in her baseline smear and her second smear, thus conferring an increased risk of 58.7 (95% CI: 10.2–∞) (Wallin *et al*, 1999).

When we compared the presence of AAV-2 DNA with HPV DNA of the same sample in the entire study material, none of the prediagnostic smear was positive for both AAV and HPV DNA while 10 biopsy case samples had both AAV and HPV DNA. None of the matched control smears were positive for both viruses. Ninety-seven samples were both negative for HPV and AAV-2 (Table 3).

## DISCUSSION

Adeno-associated virus–2 is considered a common infection worldwide in both adults and children with seroprevalence from 30 to 60% according to a study done involving individuals from Germany, Brazil and Japan (Erles *et al*, 1999). Furthermore, AAV has been postulated to be sexually transmitted as AAV DNA had been isolated from cervical samples, semen, spontaneously aborted material and gestational human trophoblasts (Atchison *et al*, 1965; Tobiasch *et al*, 1994; Kiehl *et al*, 2002).

It was suggested from previous seroepidemiological studies that AAV infection could protect women against the development of cervical cancer (Mayor *et al*, 1976). However, there were other studies that showed no significant difference between the seroprevalence for AAV among cases and controls, although lower antibody titers among cancer patients than among non-cancer patients were seen (Georg-Fries *et al*, 1984; Strickler *et al*, 1999; Smith *et al*, 2001).

DNA-based analysis on clinical samples taken from the cervix has reported inconsistent results regarding AAV and cervix cancer. Some studies detected high prevalence AAV-2 DNA in cervical samples (Han *et al*, 1996; Ahn *et al*, 2003). However, others found that AAV prevalence was not high in cervical tissue (Grossman *et al*, 1992; Strickler *et al*, 1999; Odunsi *et al*, 2000). Based on our optimised and sensitive PCR systems, a low proportion of samples were found positive are well in line with the reports of absence or low prevalence of AAV, irrespective of the nature of the samples (Grossman *et al*, 1992; Odunsi *et al*, 2000).

Experimentally, *in-vitro* co-infection of AAV and HPV may involve complex molecular/biological interactions that result in either the repression or promotion of HPV replication. *In-vitro* experiments using organotypic ‘raft’ culture system, which enable epithelial cells to reach terminal differentiation and thereby support the complete HPV life cycle indicated that when AAV-2 was present at low multiplicity of infection (MOI), HPV replication was slightly increased. The reverse was seen at high AAV MOI. Thus, suggesting that AAV may have a significant effect upon the kinetics of HPV life cycle regulation in the natural host tissue, where the exact mechanism behind the interaction effect is still largely unknown (Meyers *et al*, 2001; Agrawal *et al*, 2002).

Our study base is a population-based cohort of healthy women who did or did not develop cervical cancer on follow-up. It is a reliable study design to evaluate possible risk factors. The study design using closed cohorts with comprehensive case assessment actually has minimal risks for selection biases. All Pap smears analysed in our study were cytological normal, and the baseline for AAV DNA prevalence among case-women was low (2%), as compared to 3% among control-women. The low prevalence in our study material could be influenced by the nature of the specimen. Fixed and stained samples often present with degradation of DNA. To overcome any technical problem, the PCR systems used had been carefully optimised, validated with variable template volumes used to overcome the effect of inhibition. Furthermore, the PCR products produced were within the range suitable for amplification by PCR from archival DNA. However, biopsies contain more cells as compared to smears and that could have contributed to greater success in amplifying AAV DNA. The detection limits for the PCR systems we used were similar for both biopsy and Pap smear. Despite the higher AAV DNA prevalence among the invasive cancers (11%) as compared to the matched smears from control-women, the data suggest an association but a larger sample population utilizing fresh smears or biopsies should be ideal.

In conclusion, we found a low proportion of cervical cancer biopsies contain AAV-2 genomes. In addition, AAV DNA persistence was not detectable in our longitudinal study. Adeno-associated virus-2 infection cannot be associated with decreased or increased risk for future development of cervical cancer.

## Figures and Tables

**Table 1:** Oligonucleotide primers used in this study

**Type**	**Primer sequence**	**Position**	**Length in bp**
Pan AAV	5′-AAC TGG ACC AAT TGA AAA CTT TCC-3′		338
	5′-AAA AAG TCT TTG ACT TCC TGC TT-3′		
Non-nested AAV-2	5′-CAT CGC GGA GGC CAT AGC CC-3′	1346–1365	221
	5′-ACG GGA GTC GGG TCT ATC TG-3′	1548–1567	
Nested AAV-2	5′-ACA CCA TCT GGC TGT TTG GG-3′	1303–1323	337
	5′-GGC TGC TGG TGT TCG AAG GT-3′	1640-1620	
	5′-GAG GCC ATA GCC CAC ACT GT-3′	1353–1373	151
	5′-GAG AAT GGC TTT GGC CGA CT-3′	1483–1504	
S14	5′-CAG TGA CAT GGA CAA AAG TG-3′		150
	5′-TCG AAA GGG GAA GGA AAA GA-3′		

**Table 2:** AAV-2 prevalence among 104 cervical cancer biopsy specimens and 104 matched control cytological normal Pap smears by nested AAV-2 PCR

	**Prediagnostic smears^a^**	**Cancer biopsies/Matched smears**
Cases	2^a^	12^a^
Controls	3^a^	3^a^

a None of them represent the same patient.

**Table 3:** Comparison of the presence of AAV-2 DNA with HPV DNA in 104 cervical cancer biopsy specimens and 104 matched control smears

	**HPV+**	**HPV+**	**HPV−**	**HPV−**
	**AAV+**	**AAV−**	**AAV+**	**AAV−**
Invasive cancer biopsies	10 (10%)	70 (67%)	2 (2%)	22 (21%)
Match control smears	0 (0%)	4 (4%)	3 (3%)	97 (93%)
